# Supplementary material for: Tandem 13-Lipoxygenase Genes in a Cluster Confers Yellow-Green Leaf in Cucumber
Source: Int J Mol Sci. 2019 Jun 25;20(12):3102. doi: 10.3390/ijms20123102 (PMC6628033; doi:10.3390/ijms20123102)
Supplement: Supplementary file 1 [file ijms-20-03102-s001.zip › ijms-518265 final suppl/ijms-518265 proof 2 done suppl. .docx]

**Table S1.** Primer sequences used for qPCR and gene cloning.

| Primer | Application | Sequence (5'–3') |
| --- | --- | --- |
| SNP4G11124523 | SNP maker | Forward GAGGTGTGGCAGAATTAATCAAG  Reverse CGTCTTTCAATTCAACAATCACA |
| SNP4G11166969 | SNP maker | Forward CAGTGATTGTTGAACTGCAAGAC  Reverse GAAGAAAAGAAGATCCTTCGGTG |
| SNP4G11182070 | SNP maker | Forward GCACTTTTTGATGTTGATGGTTT  Reverse CTCACCATCCATAGGTACAGGAG |
| SNP4G11193182 | SNP maker | Forward AATTGGTCACGGTATGAATGAAT  Reverse ATTTAAGGTTTTGAATGCGGACT |
| *960* Ri1 | RNAi vector | Forward (attB1) GGGGACAAGTTTGTACAAAAAAGCAGGCT-  GCCATGGCGTCTAACATCAT  Reverse (attB2) GGGGACCACTTTGTACAAGAAAGCTGGGT-  GCGTTGGTCAGGAATGAGAC |
| *960* Ri2 | RNAi vector | Forward (attB1) GGGGACAAGTTTGTACAAAAAAGCAGGCT-  CTGAAGTTGAGAGCGGAGGA  Reverse (attB2) GGGGACCACTTTGTACAAGAAAGCTGGGT-  GGAGTGCAATGCCATCTCTG |
| *CsaUbiquitin* | qRT-PCR | Forward TTTATATGCGTTCGTGGACTGG  Reverse CTTGGTGGCTTCTCAGGGTAAT |
| *Csa4M286960* | qRT-PCR | Forward GCACTCCCATCCAATCCAC  Reverse GGGAAGAAAGATAAGTTAGGAGCA |
| *Csa4M287550* | qRT-PCR | Forward TACAGGGGTGTTGGGTTGAA  Reverse GCAAGGAGTTCACCGCAGATA |
| *Csa4M288070* | qRT-PCR | Forward TGGAGTCAGGAAGGGACAAGTT  Reverse GTGCTAAAGGGGCTAATACCG |
| *Csa4M288080* | qRT-PCR | Forward GGAGGAACGAAACGCAAGATA  Reverse TCATCGCATTACAATACTCTT |
| *cuPDS* | VIGS vector | Forward TACATGGATCCTTTGGGGCTTATCCCAAT  Reverse TACATCTCGAGTCTCATCCACTCTTGCAC |
| *960*TRV-1 | VIGS vector | Forward TACATGGATCC GCCATGGCGTCTAACATCAT  Reverse TACATCTCGAGGCGTTGGTCAGGAATGAGAC |
| *960*TRV-2 | VIGS vector | Forward TACATGGATCCCTGAAGTTGAGAGCGGAGGA  Reverse TACATCTCGAGGGAGTGCAATGCCATCTCTG |

Note: 5’-attB1, 5’-attB2 and restriction enzymes site extensions are underlined


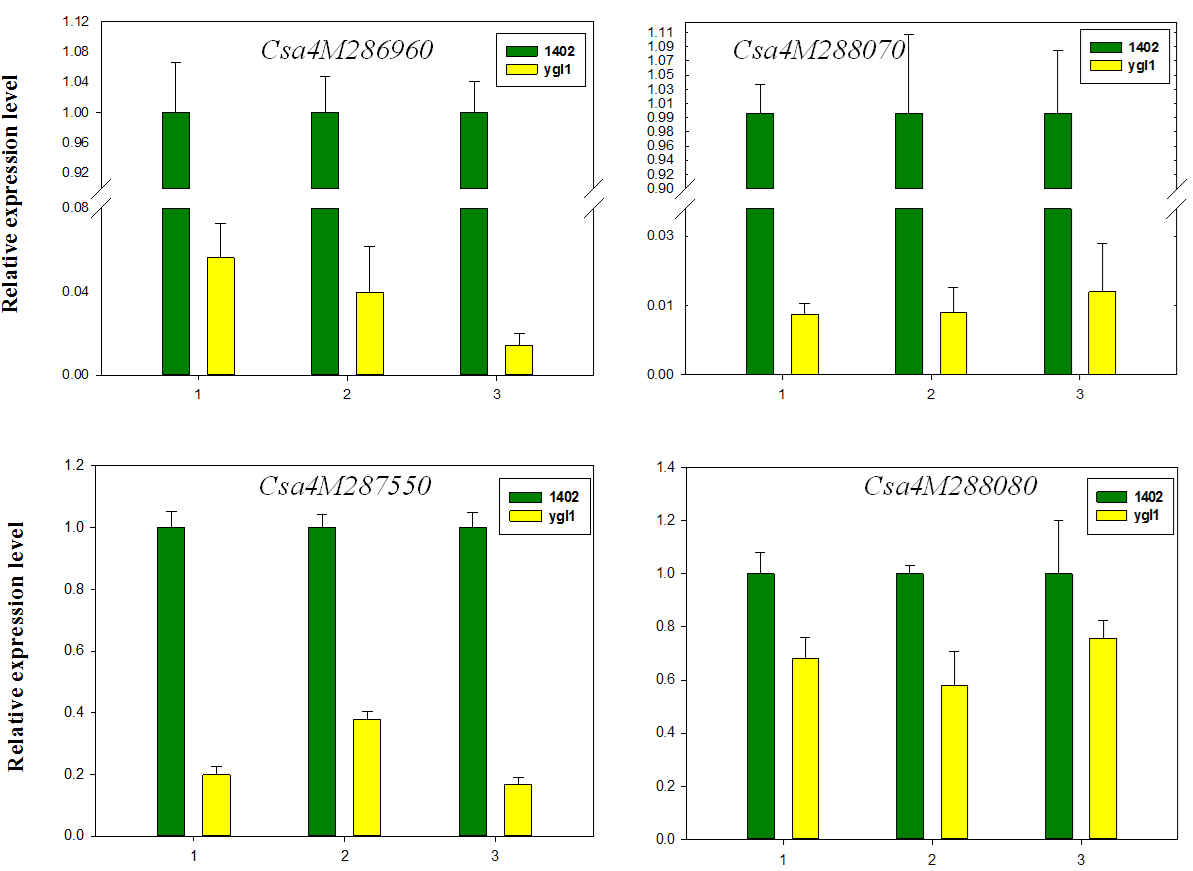


**Figure S1.** Real-time PCR expression analysis of four candidate *LOX* genes between *ygl1* and 1402. Numbers 1, 2, and 3 indicates three individual biological repeats.


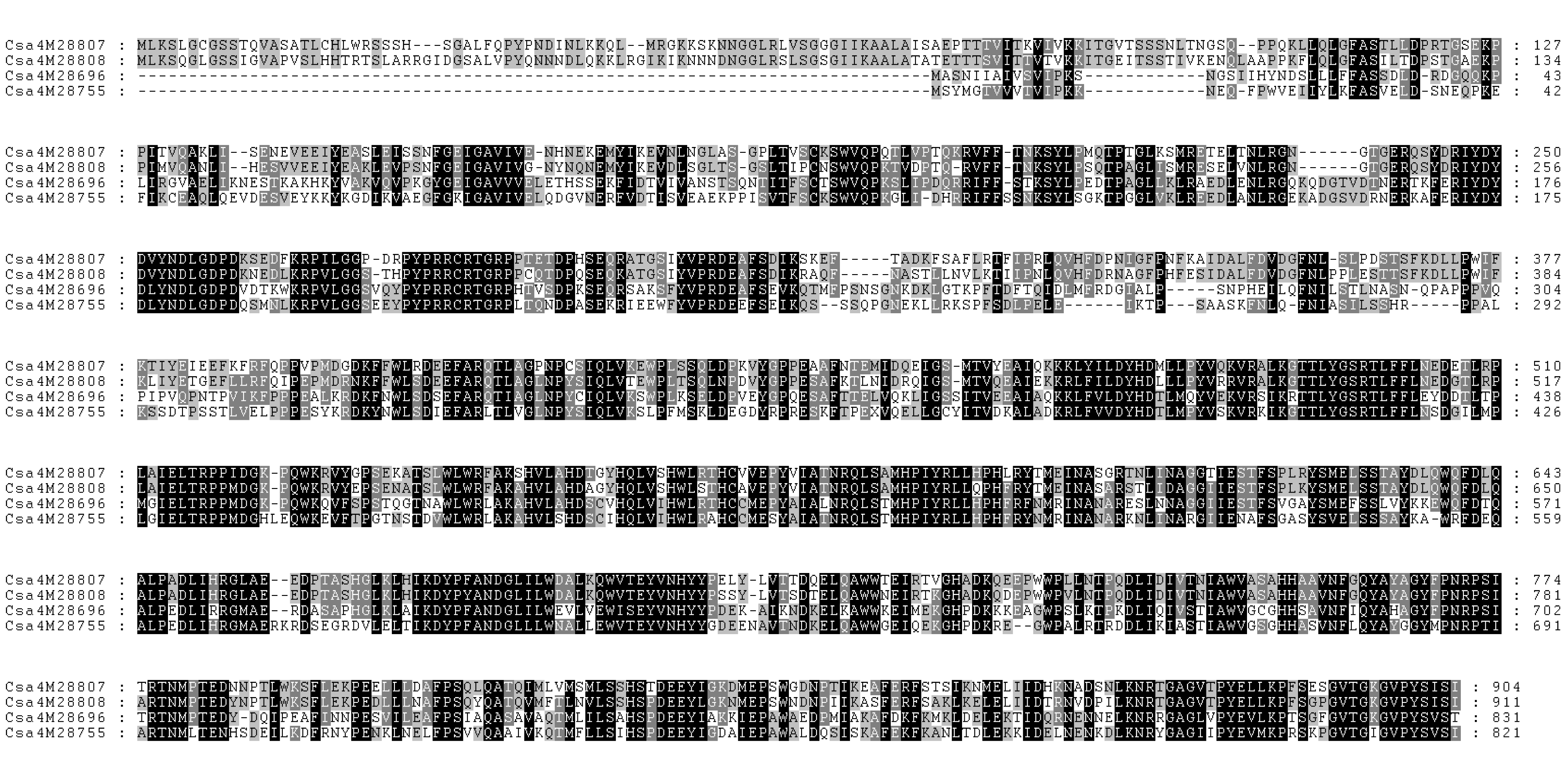


**Figure S2.** Multiple alignments in deduced amino acid of four candidate *LOX* genes.


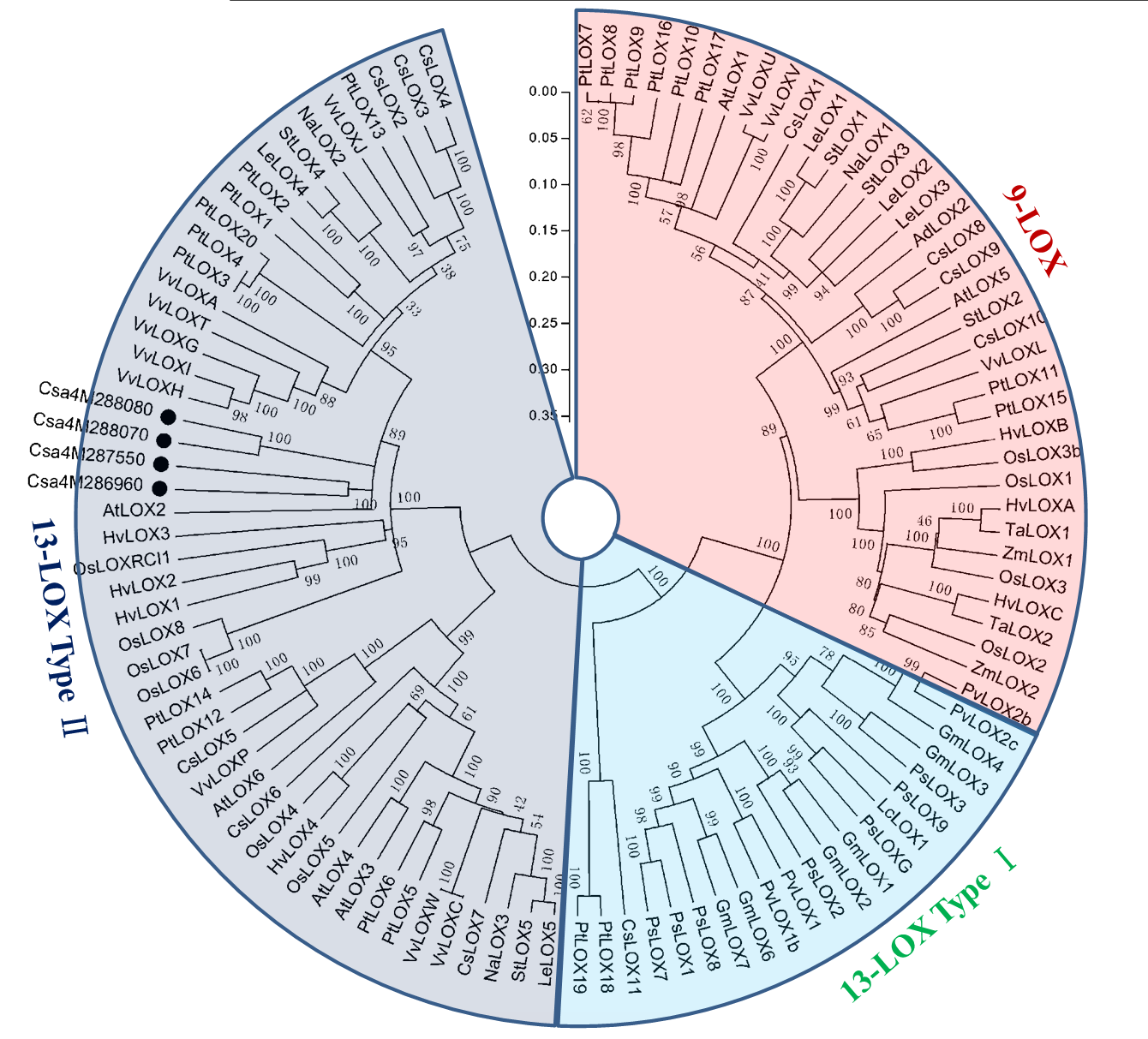


**Figure S3.** Phylogenetic analysis of four candidate LOX genes. An unrooted neighbor-joining phylogenetic tree was constructed from 102 LOX proteins from 17 plant species. A bootstrap test was set to 1000 replicates to validate tree classification confidence. The bootstrap values of the confidence levels are shown as percentages at branch nodes. The four candidate *LOX* genes are highlighted in circles.

**
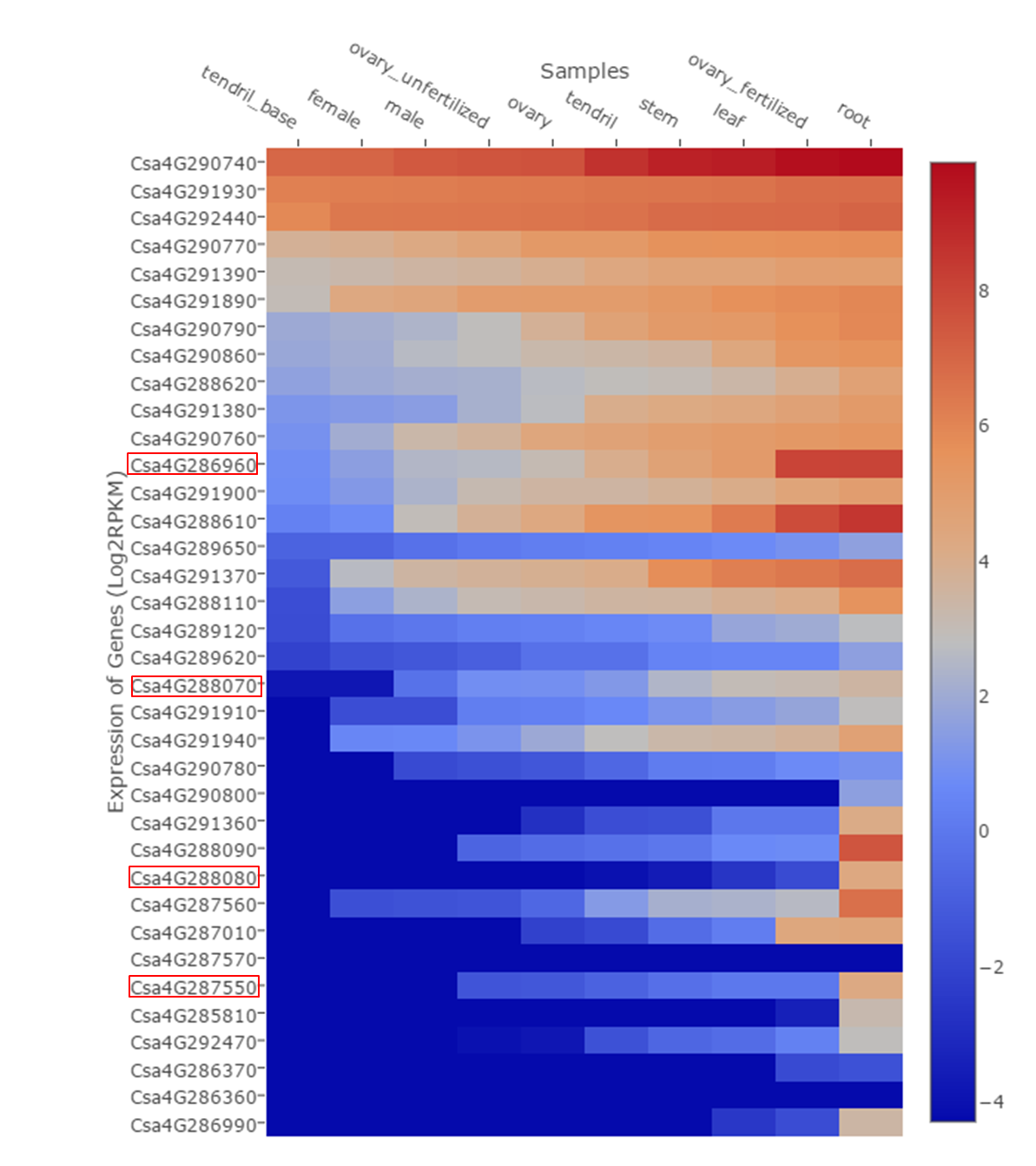
**

**Figure S4.** Heat map of four candidate *LOX* genes in various tissues. The RNA-seq expression data of different tissues were used to construct tissue expression patterns of *LOX* genes. The four candidate *LOX* genes are highlighted in red boxes.


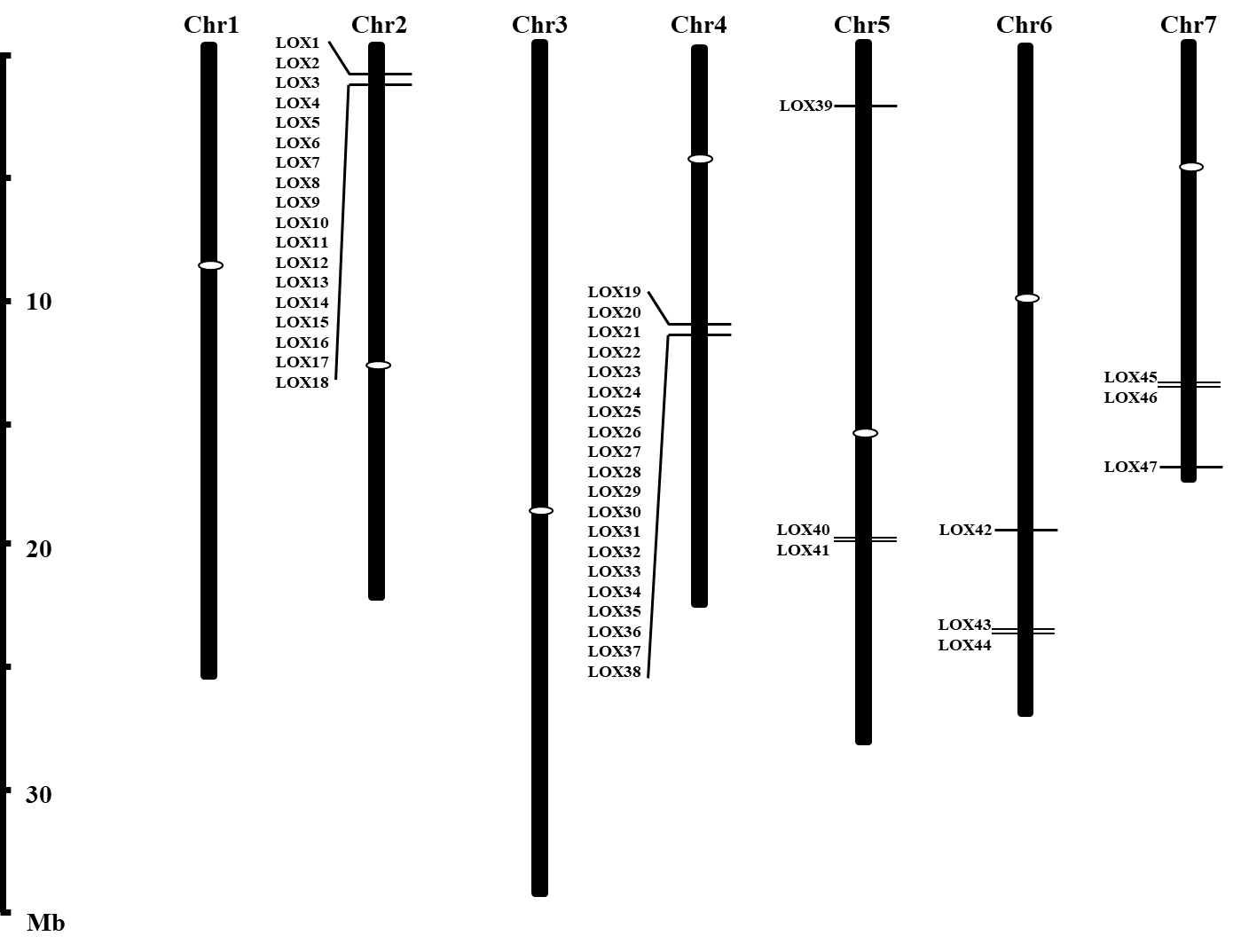


**Figure S5.** Chromosome distribution and duplication events of *LOX* genes in cucumber. Chromosomal mapping was based on the physical position (Mb) in seven cucumber chromosomes. Chromosome number is indicated at the top of each bar. Positions of the cucumber *LOX* genes in the chromosomes were obtained from Cucurbit Genomics Database (CuGenDB: http://cucurbitgenomics.org/). Black blocks indicate the centromere positions in chromosomes. Scale represents 10 Mb chromosomal distance.

© 2019 by the authors. Licensee MDPI, Basel, Switzerland. This article is an open access article distributed under the terms and conditions of the Creative Commons Attribution (CC BY) license (http://creativecommons.org/licenses/by/4.0/).
